# Contribution des diagnostics au points de service dans l’identification de la maladie à VIH avancée

**DOI:** 10.5588/pha.23.0005

**Published:** 2023-08-01

**Authors:** P. Ditondo, A. Luemba, R. Ingwe Chuy, G. Mucinya, S. Ade

**Affiliations:** 1 Médecins Sans Frontières Belgique, Kinshasa, RDC; 2 Programme National de Lutte Contre le VIH/SIDA, Kinshasa, RDC; 3 Faculté de Médecine, Université de Parakou, Parakou, Bénin

**Keywords:** diagnostic rapide, TB-LAM, CrAg, VIH avancé, point-of-care, point de service, République Démocratique du Congo

## Abstract

**CONTEXTE:**

Médecins Sans Frontières Belgique a mis en place des diagnostics au point de service (DPS) pour le dépistage précoce d’un VIH avancé, et en présence de celle-ci, d’une TB et d’une cryptococcose, dans six centres de santé (Kasai, St Ambroise, St Joseph, Libondi, Lisanga et Kimia) à Kinshasa, République Démocratique du Congo (RDC).

**OBJECTIF:**

Documenter leur contribution dans le diagnostic de ces affections.

**MÉTHODE:**

Ceci est une étude transversale rétrospective sur des adolescents et adultes VIH-positif, admis avec suspicion d’un VIH avancé. Une comparaison 2 ans avant et 2 ans après installation des DPS a été réalisée.

**RÉSULTATS:**

Au total, 745 et 887 patients étaient retenus respectivement avant et après l’installation des DPS. L’âge moyen était de 39,7 ans (déviation standard [DS] 12,04); 66% (*n* = 1 077) étaient des femmes. Les patients avec CD4 dosés étaient passés de 40,3% (*n* = 300) à 64,4% (*n* = 573) (*P < *0,001). Après l’installation des DPS, ils variaient entre 47,8% (Lisanga) et 97,1% (Kasai). La proportion d’infection à VIH avancé était comparable (*n* = 158, 52,7% vs. *n* = 288, 50,3%; *P = *0,779). Chez les patients avec un VIH avancé, la TB était dépistée chez 28,5% (*n* = 82), dont 41,5% (*n* = 34) de confirmation; la cryptococcose était dépistée chez 24,7% (*n* = 71), dont 9,9% (*n* = 7) de confirmation. Des disparités entre les centres étaient observés.

**CONCLUSION:**

Les DPS ont augmenté l’accès des patients au dosage des CD4 et au diagnostic d’un VIH avancé dans les six centres dans la RDC. Cependant des actions sont requises pour améliorer cette performance, y compris le dépistage de la TB et de la cryptococcose.

## Abstract (en)

**BACKGROUND:**

Médecins Sans Frontières Belgium installed point-of-care (POC) diagnostics for the early detection of advanced HIV disease (AHD), and in its presence, TB and cryptococcosis, in six health centres (Kasai, St Ambroise, St Joseph, Libondi, Lisanga and Kimia) in Kinshasa, Democratic Republic of the Congo (DRC).

**OBJECTIVE:**

To document their contribution to the diagnosis of these conditions.

**METHOD:**

This is a retrospective cross-sectional study of HIV-positive adolescents and adults admitted with suspected AHD. A comparison 2 years before and 2 years after installation of POC was performed.

**RESULTS:**

A total of 745 and 887 patients were included before and after POC, respectively. The mean age was 39.7 years (standard deviation [SD] 12.04); 66% (*n* = 1,077) were women. Patients with CD4 counts increased from 40.3% (*n* = 300) to 64.4% (*n* = 573) (*P* < 0.001). After the installation of POC, they ranged from 47.8% (Lisanga) to 97.1% (Kasai). The proportion of AHD was comparable (*n* = 158, 52.7% vs. *n* = 288, 50.3%; *P* = 0.779). Among patients with AHD, TB was detected in 28.5% (*n* = 82), of which 41.5% (*n* = 34) were confirmed; cryptococcosis was detected in 24.7% (*n* = 71), of which 9.9% (*n* = 7) were confirmed. Disparities between centres were observed.

**CONCLUSION:**

The POCs have increased patient access to CD4 testing and diagnosis of AHD in the six centres in DRC. However, actions are required to improve this performance, including screening for TB and cryptococcosis.

La stratégie actuellement adoptée par l’OMS pour la prise en charge des personnes vivant avec le virus de l’immunodéficience humaine (PVVIH), consistant à « tester et traiter », a permis de s’affranchir des obstacles liés à certains examens de laboratoire, en particulier le dosage systématique des lymphocytes T CD4 (CD4). Des performances encourageantes ont été enregistrées, avec la réduction des décès liés au VIH, passés de 1,1 millions en 2015 à 650 000 en 2021.^1,2^

Sans remettre en cause le bénéfice de cette stratégie, sur le terrain, sa mise en application a eu comme autre conséquence, des difficultés dans la reconnaissance précoce d’un VIH avancé, à savoir les patients ayant un taux de CD4 < 200/µl, ou admis au stade III ou IV de la classification OMS.^3^ Cependant, ces patients avec un VIH avancé, fréquents parmi les PVVIH dans nos pays d’Afrique subsaharienne, requièrent une attention particulière.^3–7^ En effet, ils ont un risque plus élevé de décès causé par des infections opportunistes (IO) sévères, comme la TB qui est la plus fréquente et la plus meurtrière, ou la cryptococcose.^4,6,8,9^ De même, le risque de survenue d’un syndrome de reconstitution immunitaire, qui complique la prise en charge du patient,^10^ est élevé en leur sein. Le dépistage précoce de cette forme sévère de la maladie, voire de certaines IO chez les patients très immunodéprimés devrait aider à réduire davantage la mortalité liée au VIH, et est fortement recommandé par l’OMS.^3^

Les diagnostics au point de service (DPS) apparaissent actuellement comme un atout certain dans le dispositif de soins.^11^ Il s’agit de tests rapides de diagnostic médical, réalisables sur place et sans délai, nécessitant peu de technicité, avec une disponibilité instantanée des résultats pour une prise de décision immédiate.^12^ Ils permettent de réaliser très rapidement un diagnostic du VIH, une numération des CD4, un dosage de la charge virale, voire la détection de certaines IO sévères (POC technologies for HIV).^12^ Des résultats positifs de l’utilisation de ces tests rapides ont été rapportés dans la littérature, en termes de gain temporaire, de rapidité de mise sous antirétroviraux (ARV), de référence des cas graves et de rétention aux soins.^13,14^

A partir de 2016, en République Démocratique du Congo (RDC), pays avec une prévalence du VIH estimée 0,7% en 2020 chez les sujets âgés de 15–49 ans,^15^ Médecins Sans Frontières (MSF) a progressivement mis en place dans six centres de santé périphériques à Kinshasa la capitale, trois types de DPS notamment ; le dosage des CD4, le diagnostic de la TB et le diagnostic de la cryptococcose. Avant cette période, ces affections étaient confirmées par des méthodes conventionnelles ; et pour de nombreux patients, le traitement était retardé, faute de résultats disponibles dans les délais, pour de multiples raisons incluant le coût de l’examen, la nécessité d’un personnel qualifié, l’obligation de se rendre à l’hôpital ou dans les cliniques universitaires disposant de laboratoires ayant un plateau technique adapté à ces maladies. Depuis la mise en place des DPS, à notre connaissance, aucun travail n’a jusque-là évalué leur contribution dans l’amélioration de l’accès au diagnostic d’un VIH avancé chez les PVVIH.

La présente a donc été initiée afin de documenter leur apport dans le diagnostic précoce d’un VIH avancé, de la TB et de la cryptococcose chez les adolescents et les adultes infectés par le VIH admis dans six centres de santé (Kasai, St Ambroise, St Joseph, Libondi, Lisanga et Kimia) à Kinshasa, République Démocratique du Congo (RDC). Les objectifs poursuivi par cette étude réalisée chez les patients nouvellement diagnostiqués 2 ans avant la mise en place des DPS (avant-DPS) et 2 ans (après-DPS) ont été de *: *1) décrire leurs caractéristiques démographiques et cliniques ; 2) comparer les proportions de taux d’accès au dosage CD4 à l’admission entre les patients admis 2 ans avant et 2 ans après d’intégration des DPS dans ces établissements de soins ; et parmi ceux-ci ; i) la proportion des patients ayant un taux de CD4 <200 cellules/µl ; ii) déterminer chez les patients diagnostiqués avec un VIH avancé après-DPS : 1) la proportion des patients qui ont eu un dépistage de la TB et de la cryptococcose à l’admission ; et parmi eux, i) déterminer la proportion de cas confirmés.

## TYPE, POPULATION ET MÉTHODE D’ÉTUDE

### Type d’étude

Il s’agissait d’une étude transversale comparant des données rétrospectives collectées 2 ans avant puis 2 ans après la mise en place des DPS. Plus précisément, la période d’étude s’étend de mars 2014 à juillet 2022.

### Cadre

Le soutien MSF Belgique pour les six centres de santé inclus dans l’étude a commencé en mars 2016 pour Kimia, en août 2016 pour Lisanga, en mars 2018 pour Libondi, en septembre 2019 pour Maternité Kasai, en mars 2020 pour Saint Ambroise et en juillet 2020 pour Saint Joseph. Implantés en périphérie de la ville, ils desservent une population vulnérable, confrontée à des ressources limitées et des difficultés d’accès aux soins de qualité. Ils ont un paquet minimum d’activités médicales, incluant les consultations ambulatoires, les activités de laboratoire, la maternité, la prévention de la transmission mère-enfant, la prise en charge des PVVIH et celle des tuberculeux. Les patients souffrant d’un VIH avancé et dans un état critique sont référés vers l’hôpital général. Le personnel soignant est composé d’infirmiers polyvalents, d’aides-soignants, de sages femmes. Ces centres réalisent en moyenne mensuellement 200 consultations VIH.

Dans chaque centre inclus, la mise en place des tests était systématiquement précédée d’une formation d’une semaine en moyenne sur l’utilisation des appareils et des intrants. Après celle-ci, l’Analyser Pima (Abbott, Chicago, IL, USA) pour le compte des CD4 a été intégré, suivi de TB lipoarabinomannane (TB-LAM) pour la TB et enfin cryptococcoque dans le sang (CrAg sérique) pour le diagnostic de la cryptococcose. Par la suite, une évaluation régulière a été organisée tout au long de l’appui MSF, afin d’assurer la qualité et de la validité des résultats.

### Initiation de la prise en charge des nouvelles PVVIH

Une fois le diagnostic d’infection à VIH confirmé, le patient est consulté par un infirmier qui établit un bon d’examen pour la réalisation des tests DPS. Des échantillons de sang et d’urine sont ensuite prélevés au laboratoire. Les CD4 sont dosés en premier lieu par un analyseur Pima. Lorsqu’ils sont inférieurs à 200 cellules/µl, les urines sont analysées par TB-LAM test et le reste du prélèvement sanguin sert à la recherche de l’antigène spécifique du cryptococcoque dans le sang (CrAg serique).^16–19^ Les résultats de tous les examens sont disponibles en 45 min en moyenne. Le patient rencontre à nouveau l’infirmier pour la suite de la prise en charge. Ces tests sont parfois réalisés par l’infirmier lui-même pendant les moments de faible fréquentation. La prise en charge de ces patients infectés par le VIH est gratuite dans ces centres.

### Population d’étude

Les PVVIH adolescents (10–17 ans) ou adultes (⩾18 ans) nouvellement diagnostiqués avant et après installation des DPS dans ces centres étaient inclus dans l’étude. Les informations sur les différentes périodes selon le centre sont récapitulées dans le Tableau 1.

**TABLEAU 1:** Périodes d’étude choisies dans les six centres de santé soutenus par MSF, Kinshasa, RDC

Centre de santé	Début de la période de l’étude*	Date de début du soutien de MSF	Fin de la période d’étude^†^
Kimia	01 March 2014	01 March 2016	31 March 2018
Lisanga	01 August 2014	01 August 2016	31 August 2018
Libondi	01 March 2016	01 March 2018	31 March 2020
Maternité Kasaï	01 September 2017	01 September 2019	30 September 2021
Saint Ambroise	01 March 2018	01 March 2020	31 March 2022
Saint Joseph	01 July 2018	01 July 2020	31 July 2022

*2 ans avant soutien MSF.

^†^2 ans après début soutien MSF pour comparaison avec la période de 2 ans avant soutien MSF.

MSF = Médecins Sans Frontières ; RDC = République Démocratique du Congo.

### Principales variables

Les principales variables colligées étaient les caractéristiques sociodémographiques, cliniques, les informations relatives à la réalisation du dosage des CD4, en particulier le moment de réalisation après le diagnostic et le résultat obtenu ; et pour les patients avec un VIH avancé dans la période après-DPS, des informations supplémentaires sur la date de réalisation d’une recherche de TB par TB-LAM et de cryptococcose par CrAg.

### Collecte et traitements des données

Les informations sont disponibles dans un système électronique de gestion des données des patients (Tier.Net). Il s’agit d’un système préalablement renseigné par des encodeurs à partir des dossiers physiques des patients, et régulièrement validé par un gestionnaire des données. En cas de constatation d’une aberration ou d’une discordance, le gestionnaire recourt au dossier médical individuel pour une éventuelle correction. Les informations sur les variables ont été par la suite exportées dans un fichier Excel (Microsoft, Redmond, WA, USA). Les données manquantes sur la feuille Excel étaient autant que possible complétées par l’investigateur principal, à partir des dossiers physiques.

### Analyse statistique

Après correction de la base, tous les identifiants des patients ont été supprimés. Les informations ont été ensuite analysées dans EpiData Analysis v2.2.3.187 (EpiData Association Odense, Denmark). Les variables quantitatives ont été décrites en utilisant les moyennes (déviation standard [DS]), les variables qualitatives par les fréquences et les pourcentages. Les variables qualitatives ont été comparées en utilisant le test du χ^2^ (ou le test de Fisher ou de Yates quand les effectifs sont <5).

### Considérations éthiques

L’étude a été approuvée par le Comité d’Ethique de l’Université de Kinshasa (N°ESP/CE/20/2022). De même, cette recherche remplissait les critères d’exemption fixés par le comité d’éthique de Médecins Sans Frontières (MSF Ethics Review Board ; Génève, La Suisse) pour les analyses à posteriori des données cliniques collectées de manière routinière, et ne nécessitait donc pas d’examen MSF ERB. Elle a été menée avec l’autorisation du directeur médical du centre opérationnel. Il convient de noter que tous les patients soignés par MSF ont donné leur consentement médical ; ceci étant une étude rétrospective, il n’était pas nécessaire d’obtenir le consentement des patients à ce moment-là.

## RÉSULTATS

Au total, 1 632 patients nouvellement diagnostiqués ont été admis dans les six structures, 745 dans la période avant-DPS et 887 dans celle après-DPS. Ces patients avaient un âge moyen de 39,7 ans (DS 12,04) ; les femmes représentaient 66% de l’effectif ; le traitement ARV était initié chez 1 426 (87,4%) des patients, parmi lesquels 607 (42,6%) étaient au stade 3 de l’OMS. Deux ans après l’installation des DPS, les proportions de patients rapportés « perdus de vue » (58,6% vs. 66,2% ; *P < *0,001) et « décédés » (3,2% vs. 5,2% ; *P = *0,01) avaient significativement diminué comme à ceux de la période avant DPS (Tableau 2). La proportion ayant eu un dosage des CD4 était passée de 40,3% avant-DPS à 64,4% après-DPS (*P < *0,001). Des taux significativement plus élevés ont été rapportés de certains centres de santé depuis l’installation des DPS. Il s’agit de Kasai (*P < *0,001), Saint Ambroise (*P < *0,001) et Saint Joseph (*P < *0,001), avec des proportions variant entre 84,2% à Saint Joseph et 97,1% à Kasai. Ce taux a connu une tendance à la baisse à Lisanga (63,7% vs. 47,8% ; *P = *0,036) et a Kimia (75,6% vs. 60,9% ; *P = *0,058). Quant à la proportion de nouveaux patients diagnostiqués avec un VIH avancé, elle était de 52,7% avant-DPS et de 50,3% après-DPS (*P = *0,779) (Tableau 3). Depuis la mise en place des DPS parmi les patients avec un VIH avancé, 82 (28,5%) avaient eu la recherche de la TB par TB-LAM dans les urines. La fréquence du dépistage variait entre 87,5% à Kasai et 0% à Libondi ; la TB était confirmée chez 34 (41,5%). Quant à la cryptococcose, le dépistage était effectué chez 71 (24,7%), patients avec des fréquences variant entre 63,6% à St Ambroise et 0% à Lisanga, et une confirmation du diagnostic chez 7 (9,9%) (Tableau 4).

**TABLEAU 2:** Caractéristiques démographiques et cliniques et devenir des patients séropositifs pour le VIH nouvellement admis dans les six structures, 2 ans avant et 2 ans après la mise en place des diagnostics au point de service

	Tout patient *n* (%)	2 ans avant DPS *n* (%)	2 ans après DPS *n* (%)
Sexe			
Masculin	555 (34)	246 (33)	309 (34,8)
Féminin	1 077 (66)	499 (67)	578 (65,2)
Tranche d’âge, ans			
10–17	59 (3,6)	22 (3)	37 (4,2)
⩾18	1 573 (96,4)	723 (97)	850 (95,8)
Centre de santé de prise en charge			
Kasai	62 (3,8)	27 (3,6)	35 (3,9)
Saint Ambroise	87 (5,3)	24 (3,2)	63 (7,1)
Saint Joseph	171 (10,5)	95 (12,8)	76 (8,6)
Lisanga	398 (24,4)	168 (22,6)	230 (25,9)
Libondi	456 (27,9)	234 (31,4)	222 (25,0)
Kimia	458 (28,1)	187 (26,4)	261 (29,4)
Statut par rapport à la mise sous			
ARV			
Mis sous ARV	1 426 (87,4)	699 (93,8)	727 (82,0)
ARV non débuté	206 (12,6)	46 (6,2)	160 (18,0)
Stade OMS à la mise sous ARV*			
Stade 1	355 (24,9)	88 (12,6)	267 (36,7)
Stade 2	346 (24,3)	254 (36,3)	92 (12,7)
Stade 3	607 (42,6)	314 (44,9)	293 (40,3)
Stade 4	111 (7,8)	41 (5,9)	70 (9,6)
Stade non renseigné	7 (0,5)	2 (0,3)	5 (0,7)
Devenir des patients*^†^			
Vivant	380 (26,6)	147 (21)	233 (32)
Décédé	59 (4,1)	36 (5,2)	23 (3,2)
Perdu de vue	890 (62,4)	464 (66,4)	426 (58,6)
Transféré	97 (6,8)	52 (7,4)	45 (6,2)
Total	1 632	745	887

*Pourcentage calculé à partir des patients mis sous ARV 2 ans avant et 2 ans après DPS.

^†^Evaluation lors de la mise en œuvre de l’étude.

DPS = diagnostic au point de service ; ARV = antirétroviral.

**TABLEAU 3:** Taux de réalisation des CD4 et de diagnostic d’un VIH avancé chez les patients séropositifs pour le VIH nouvellement admis dans les six structures, 2 ans avant et 2 ans après la mise en place des DPS

	2 ans avant DPS			2 ans après DPS			*P* *
	Total *n* (%)	CD4 fait *n* (%)	CD4 <200 céllules/µl *n* (%)	Total *n*	CD4 fait *n* (%)	CD4 <200 céllules/µl *n* (%)	
Kasai	27	0 (0)	0 (0)	35	34 (97,1)	16 (47,1)	<0,001
St Ambroise	24	1 (4,2)	0 (0)	63	57 (90,5)	22 (38,6)	<0,001
St Joseph	95	0 (0)	0 (0)	76	64 (84,2)	28 (43,8)	<0,001
Libondi	234	43 (18,4)	17 (39,5)	222	149 (67,1)	85 (57,0)	<0,001
Lisanga	168	107 (63,7)	58 (54,2)	230	110 (47,8)	53 (48,2)	0,036
Kimia	197	149 (75,6)	83 (55,7)	261	159 (60,9)	84 (52,8)	0,058
Tout centre	745	300 (40,3)	158 (52,7)	887	573 (64,6)	288 (50,3)	<0,001

*Comparaison entre les proportions de patients ayant eu le dosage des CD4 2 ans avant et 2 ans après l’installation des DPS.

DPS = diagnostic au point de service.

**TABLEAU 4:** Taux de réalisation du dépistage de la TB et de la cryptococcose chez les patients souffrant d’un VIH avancé diagnostiqués depuis la mise en place des diagnostics au point de service dans les six centres

	CD4 <200 céllules/µl *n*	TB		Cryptococcose	
		TB-LAM fait *n* (%)	Diagnostic confirmé *n* (%)	CrAg fait *n* (%)	Diagnostic confirmé *n* (%)
Kasai	16	14 (87,5)	0 (0)	9 (56,3)	0 (0)
St Ambroise	22	14 (63,6)	8 (57,1)	14 (63,6)	0 (0)
St Joseph	28	13 (46,4)	10 (76,9)	7 (25)	4 (57,1)
Libondi	85	31 (36,5)	7 (22,6)	41 (48,2)	3 (7,3)
Lisanga	53	0 (0)	0 (0)	0 (0)	0 (0)
Kimia	84	10 (11,9)	9 (90)	0 (0)	0 (0)
Tout centre	288	82 (28,5)	34 (41,5)	71 (24,7)	7 (9,9)

TB-LAM = TB-lipoarabinomannane ; CrAg = cryptococcoque dans le sang.

## DISCUSSION

L’objectif du présent travail était de documenter l’apport des DPS dans la prise en charge de l’infection à VIH, en particulier la détection du VIH avancé, et parmi ces patients sévèrement immunodéprimés, celle de la TB et de la cryptococcose, deux parmi les IO les plus sévères.

Dans l’ensemble, il a été mis en évidence une augmentation significative de la réalisation de la numération des CD4 dans les six centres. Avant-DPS, quatre patients sur 10 étaient testés. Après-DPS, plus de 60% des patients ont pu être testés. Cette progression a été particulièrement remarquable dans certains centres où, avant la mise en place des tests, le dosage des CD4 était quasi-inexistent. Il s’agissait des centres de santé de Kasai, de Saint Ambroise et de Saint Joseph.

Ce résultat, quoiqu’encourageant interpelle aussi. Dans un travail provenant de la même ville, mais réalisé dans un hôpital de référence, des résultats meilleurs ont été rapportés par les auteurs, après implémentation d’un nouveau guide, avec une fréquence de numération des CD4 passée de 63% à 99%.^9^ Dans notre travail, le taux de CD4 n’était pas dosé chez près de quatre patients sur 10, avec des proportions variables selon les centres. Après investigation auprès des prestataires, certaines raisons ont été évoquées. Elles incluent la survenue de ruptures de stock en intrants ; la mauvaise qualité de certains prélèvements occasionnant des allées et venues, sources de découragement pour certains patients ; et le déni du diagnostic de l’infection à VIH chez d’autres, rendant difficile la poursuite de la prise en charge. A ces raisons, s’ajoutent aussi quelquefois des restrictions horaires lors de la réception des prélèvements au laboratoire.

Par ailleurs, nous avons été surpris par la diminution de la réalisation de la numération des CD4 à Lisanga et à Kimia. Des explications fournies, elle serait la conséquence de la mise en application de la stratégie « tester et traiter », rendant désormais non obligatoire le dosage des CD4 avant la mise sous ARV.

Quant à la proportion de VIH avancé diagnostiquée, malgré l’augmentation du nombre de patients ayant eu un dosage des CD4, elle est restée quasi-similaire entre les deux périodes étudiées. Nous pensons qu’elle pourrait augmenter si tous les patients étaient testés. Dans tous les cas, ces patients avec un VIH avancé représentent la moitié des sujets testés. Cette proportion élevée appelle à une intensification de la sensibilisation au sein de la communauté pour un dépistage précoce de l’infection à VIH, et un traitement rapide pour prévenir l’effondrement de l’immunité.

Chez les patients avec un VIH avancé, la TB a pu être dépistée chez près de trois d’entre eux sur 10 par TB-LAM ; mais aucun test n’a été réalisé à Libondi. D’eux, la maladie était confirmée chez un peu plus de 40%. Concernant la cryptococcose, elle a été recherchée chez près d’un patient sur quatre, mais aucun test réalisé à Lisanga n’était rapporté. La confirmation du diagnostic était obtenue chez près un patient sur 10, comparable au taux rapporté dans un autre travail à Addis Abeba, Éthiopie (9,1%).^20^ Les raisons de la faible proportion du taux de dépistage de ces IO dans notre contexte ne sont pas pleinement connues. Après investigation, elle pourrait se justifier par le fait que la grande majorité des patients était référée vers les hôpitaux soit pour une meilleure prise en charge d’un tableau clinique préoccupant, soit pour le suivi du VIH avancé, conformément aux recommandations.^3^ A ce propos, selon les investigations, c’est ce qui justifie l’absence totale de dépistage de ces IO à Lisanga, où le circuit de détection de ces affections a été totalement orienté vers le centre hospitaliser de Kabinda entièrement supporté par MSF, afin de palier à des difficultés de ressources humaines. De plus, d’autres, avant leur admission, étaient déjà diagnostiqués pour ces IO et mis sous traitement spécifique. Cependant, la non adaptation des outils de suivi des patients avec un VIH avancé pour documenter systématiquement la réalisation du TB-LAM et du CrAg pourrait aussi émousser le réflexe de demande de ces tests et devrait faire l’objet de réflexion. Dans tous les cas, il importe de bien comprendre tous les déterminants de cette faible proportion rapportée, pour éventuellement des actions correctrices.

Les points forts de cette étude sont qu’elle rapporte les résultats d’une action menée dans des conditions de travail normales, qu’elle a inclus un grand nombre de patients dans des centres de santé périphériques desservant une population vulnérable et qu’elle a inclus presque tous les nouveaux patients admis dans le centre pendant la période de l’étude (ce qui réduit les biais liés aux fluctuations de l’échantillonnage). Cependant, l’étude présente certaines limites. Il s’agit des lacunes communes aux études rétrospectives, avec certaines données manquantes, qui ont été complétées autant que possible à partir des dossiers physiques. En outre, certaines données supplémentaires qui auraient été utiles pour évaluer la contribution des DPS n’étaient pas disponibles, incluant par exemple, des données sur le rapport coût-efficacité et l’acceptabilité du test par rapport aux méthodes conventionnelles. Cependant, d’autres recherches ont abordé ces aspects et rapporté que l’utilisation des DPS était rentable et acceptée par la communauté et la majorité des patients.^13,14,21^ De même, l’influence des DPS sur la survie, dont les déterminants sont multifactoriels, n’a pas pu être évaluée.

Ce travail suggère quelques implications opérationnelles. D’abord la fréquence de réalisation des CD4 a, dans une certaine proportion, augmenté, malgré les difficultés rencontrées sur le terrain, et pourrait faire considérer leur extension vers d’autres centres de prise en charge des PVVIH. Ensuite, les insuffisances rapportées méritent des actions correctrices pour parvenir améliorer les performances actuelles. Elles pourraient viser la réduction des prélèvements de mauvaise qualité à travers une remise à niveau du personnel ou une amélioration des conditions de conservation ; celle des ruptures de stock des intrants. Des échanges avec le personnel des laboratoires pourraient déboucher sur une plus grande flexibilité lors du dépôt des prélèvements. Des améliorations des outils de suivi des patients avec un VIH avancé pourraient être apportées pour mieux aiguiser le réflexe de demande du dépistage de la TB et de la cryptococcose. Enfin, l’extension à d’autres DPS le diagnostic de l’infection à VIH et la mesure de la charge virale devraient aider à améliorer la lutte contre cette pandémie sur le continent.^12,22^

En conclusion, l’installation des DPS dans les six centres a amélioré le taux de réalisation des CD4, même si la proportion de patients ayant un VIH avancé diagnostiquée n’a pas augmenté. Le dépistage systématique de la TB et de la cryptococcose a été rapporté chez approximativement le quart des patients avec un VIH avancé. Des actions sont nécessaires pour améliorer ces performances et la prise en charge des PVVIH.

### Remerciements

Les auteurs remercient à P Isaakidis et W Ngwa pour leur contribution dans la révision du protocole ; D Obach dans l’apprêtement de la base de données ; à G Nsiku du Programme National de Lutte contre le SIDA pour avoir rendu disponibles les bases analysées ; à toute l’équipe de décentralisation du projet SIDA pour avoir aidé à la complétude des données manquantes ; et au projet SIDA pour les différentes facilitations.

Cette recherche a été menée dans le cadre de l’Initiative de recherche opérationnelle et de formation structurée (SORT-IT), un partenariat mondial dirigé par le Programme spécial de recherche et de formation sur les maladies tropicales de l’Organisation mondiale de la santé (OMS/TDR). Le modèle est basé sur un cours élaboré conjointement par l’Union internationale contre la tuberculose et les maladies respiratoires (L’Union) et Médecins Sans Frontières (MSF/Doctors Without Borders). Le programme spécifique, SORT-IT, qui a donné lieu à cette publication a été organisé par MSF spécifiquement pour la recherche en langue français. Le programme a été financé par La Fondation Veuve Emile Metz-Tesch, Luxembourg. Le financeur n’a joué aucun rôle dans la conception de l’étude, la collecte et l’analyse des données, la décision de publier ou la préparation du manuscrit.
